# Toxicity of the New Psychoactive Substance (NPS) Clephedrone (4-Chloromethcathinone, 4-CMC): Prediction of Toxicity Using In Silico Methods for Clinical and Forensic Purposes

**DOI:** 10.3390/ijms25115867

**Published:** 2024-05-28

**Authors:** Kamil Jurowski, Łukasz Niżnik

**Affiliations:** 1 Laboratory of Innovative Toxicological Research and Analyses, Institute of Medical Sciences, Medical College, Rzeszów University, Al. mjr. W. Kopisto 2a, 35-959 Rzeszów, Poland; 2 Department of Regulatory and Forensic Toxicology, Institute of Medical Expertise, Łódź, ul. Aleksandrowska 67/93, 91-205 Łódź, Poland; lukasz.niznik@iem.gov.pl

**Keywords:** 4-CMC, 4-chloromethcathinone, clephedrone, drug toxicity, new psychoactive substance (NPS), in silico toxicology

## Abstract

This study reports the first application of in silico methods to assess the toxicity of 4-chloromethcathinone (4-CMC), a novel psychoactive substance (NPS). Employing advanced toxicology in silico tools, it was possible to predict crucial aspects of the toxicological profile of 4-CMC, including acute toxicity (LD_50_), genotoxicity, cardiotoxicity, and its potential for endocrine disruption. The obtained results indicate significant acute toxicity with species-specific variability, moderate genotoxic potential suggesting the risk of DNA damage, and a notable cardiotoxicity risk associated with hERG channel inhibition. Endocrine disruption assessment revealed a low probability of 4-CMC interacting with estrogen receptor alpha (ER-α), suggesting minimal estrogenic activity. These insights, derived from in silico studies, are critical in advancing the understanding of 4-CMC properties in forensic and clinical toxicology. These initial toxicological findings provide a foundation for future research and aid in the formulation of risk assessment and management strategies in the context of the use and abuse of NPSs.

## 1. Introduction

From an applied toxicology perspective, 4-chloromethcathinone can be classified as a new psychoactive substance (NPS) [1]. This NPS, also known as 4-CMC or clephedrone, is a synthetic stimulant drug of the cathinone class [2,3], bearing a structure similar to that of many previously identified cathinones [4]. It would seem that this substance should be well described in the professional literature, but surprisingly most of the data are found in grey literature (Internet forums, such as ‘hyperreal.info’ from Poland, October 2015, and ‘Erowid’ from the U.S.A., April 2016). To date, no clinical data on this compound are available. Only a few studies have been published that focused on its determination in biological samples by GC-MS or CE [3] and its toxicokinetics, involving the identification of metabolites in human liver microsomes and investigations of metabolic pathways [5]. Furthermore, limited information is available regarding the physicochemical properties of this substance. The existing knowledge is briefly summarized in Table 1, based on available sources.

This table aims to collate and present the sparse data that were gathered from various databases [6,7] (PubChem) (CompTox Dashboard 2023). However, it is important to note that due to the relatively obscure nature of this substance, comprehensive and detailed information may not be readily accessible or well documented in the scientific literature. It is structurally very close to 4-chloroethcathinone (4-CEC). An isomer of 4-CMC, differing only in the position of the chlorine atom substituted on the benzene ring, is 3-chloromethcathinone (3-CMC) [8]. 4-CMC also shares structural similarities with mephedrone (4-MMC) and flephedrone (4-FMC). The main distinction lies in the replacement of the chloro group at position 4 [9]. Like other synthetic cathinones, 4-CMC was designed to mimic the effects of natural cathinone compounds found in the khat plant. However, synthetic versions, such as 4-CMC, are often much more potent and can have unpredictable effects. The position of the chlorine atom in isomers (such as 3-CMC and 4-CMC) can significantly impact the pharmacological properties and toxicity. Therefore, from a toxicological perspective, the analysis of the molecular structure of 4-chloromethcathinone (4-CMC) is crucial to understanding its potential toxicological effects. However, surprisingly, to this day there are no studies taking into account quantitative structure–activity relationships (QSARs).

**Figure 1:**
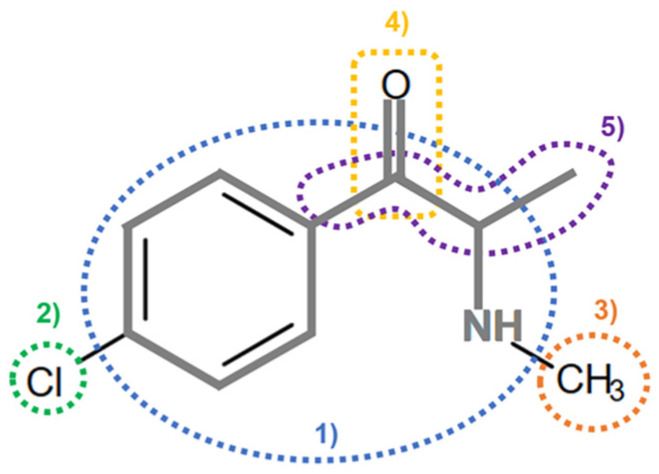
Chemical structure analysis of 4-chloromethcathinone (4-CMC, clephedrone), relevant from a toxicological point of view, with important structural fragments, indicated as (1)–(5), linked to cathinone, whose structure is shown in grey.

On the basis of its chemical architecture, it is possible to define a few important fragments, summarized in Figure 1 and described below as follows:

(1) A basic β-keto phenethylamine structure: 4-CMC possesses a basic β-keto phenethylamine structure; this structure is similar to that of amphetamines and plays a significant role in toxicology due to its impact on the interactions of the substance with various neurotransmitter systems in the body, including dopaminergic, serotonergic and adrenergic systems.
(2) The position of the chlorine atom: the presence of a chlorine atom in the para position (fourth position) of the phenyl ring (hence, ‘4-chloro’) is a key feature; this modification can affect the lipophilicity of the compound, how it crosses the blood–brain barrier, and its overall metabolic stability, which are important for determining its toxicological profile.
(3) A methyl group linked to the nitrogen atom (amino group): the term ‘meth’ in 4-CMC indicates a methyl group attached to the nitrogen atom in the amine group; this modification is common in many stimulants and can influence the pharmacokinetics of the compound, including absorption, distribution, metabolism, and excretion (ADME), often increasing the lipid solubility of the compound and potentially leading to a faster onset of action.
(4) A ketone group: The presence of a ketone group, as indicated by the suffix ‘one’ in the name, is characteristic of cathinones; in 4-CMC, this ketone group is linked to the first carbon of the ethyl chain. The ketone functionality is crucial for the substance’s reactivity and interaction with enzymatic systems in the body, affecting its metabolic pathways and potential toxicity.
(5) An ethyl chain: the ethyl chain that links the amine group and the aromatic ring in 4-CMC affects the spatial configuration; this can influence how the drug binds to neural receptors, affecting its potency and the nature of its toxic effects.

The structural analysis of 4-chloromethcathinone (4-CMC, clephedrone) provides a basis for understanding the toxic properties of this substance.

The significance of these in silico toxicology studies in the context of forensic and clinical toxicology is multiple. First, they offer for the first time groundbreaking insights into the toxicological profile of a novel psychoactive substance, which previously lacked scientific data. This paves the way for the advancement of in silico toxicology investigations in this field. Secondly, the results can be invaluable for medicolegal expert opinions, providing evidence-based information on the substance’s potential health risks, which is critical for legal and medical decision-making processes, given, especially, the varying legal classifications and control measures for this substance across different jurisdictions, which are summarized in Table 2.

## 2. Results

### 2.1. Qualitative In Silico Methods

#### Acute Toxicity/Eye and Skin Irritation

Table 3 displays the qualitative evaluations of 4-CMC acute toxicity following oral, dermal, and inhalation exposure, as well as its potential for skin and eye irritation. These forecasts were generated using specific tools, specifically, STopTox (https://stoptox.mml.unc.edu/, accessed on 30 April 2023), admetSAR 3.0, and ADMETlab 2.0.

The compound was determined by STopTox to be toxic for oral and dermal administration, with a confidence level of, respectively, 82% and 67%. However, for the inhalation route of administration, the prediction did not show toxicity, with a probability level of 50%. Furthermore, STopTox predicts the contribution of structure fragments to the toxicity profile and the applicability domain. Structural fragments that increase toxicity are marked in red, while those that decrease toxicity are marked in green [10]. The admetSAR application accurately categorized the compound’s acute oral toxicity as falling into the third category according to the U.S. EPA guidelines (with Category I toxicity being up to 50 mg/kg, Category II, between 50 and 500 mg/kg, Category III, between 500 and 5000 mg/kg, and Category IV, greater than 5000 mg/kg), with 85.47% prediction probability. AdmetSAR does not provide a qualitative classification profile for dermal acute toxicity and inhalation acute toxicity. Additionally, negative effects on eye irritation were demonstrated with a prediction probability of 58.38%, and positive effects on skin irritation with a probability of 53.28%. In addition, the probability of oral acute toxicity was estimated in ADMETlab to be 65.1%.

### 2.2. Quantitative In Silico Methods

#### 2.2.1. Acute Toxicity

The LD_50_, representing the dose at which 50% of tested animals experience death after the administration of a substance, is a pivotal measure in toxicology, reflecting a substance’s potential to induce various acute reactions, including death, in experimental subjects [11]. Widely recognized and utilized in toxicological studies, the LD_50_ offers vital insights into a substance’s immediate toxic impact after brief exposure to it. The acute toxicity module efficiently predicts the LD_50_ values for compounds tested in rodents, covering different administration routes. Table 4 provides a succinct overview of the findings regarding 4-CMC.

Differences in LD_50_ values among various species and administration methods highlight the unique toxicological characteristics of the compound, essential for assessing its safety and potential hazards. Additionally, the reliability index (RI) accompanying each LD_50_ value provides further clarity by indicating the confidence level associated with the measurement, aiding in data interpretation [12]. As for Percepta, most of the obtained data had moderate reliability, with the highest one measured for mouse toxicity using the intraperitoneal administration route (LD_50_ value of 260 mg/kg bw, reliability index of 0.83). In the case of the TEST results, the consensus method was the most reliable according to authors [13,14] and calculated an LD_50_ value of 242.07 mg/kg bw. The hierarchical method provided an LD_50_ value of 169.97 mg/kg bw, whereas the nearest-neighbor method indicated the highest LD50 value for 4-CMC, corresponding to 345.38 mg/kg bw. In addition, the VEGA and ProTox methods were also utilized to predict the acute toxicity of 4-CMC, and provided the values of 201.85 mg/kg bw and 340.00 mg/kg bw, respectively. The estimated prediction accuracy for ProTox was 70.97%.

#### 2.2.2. Health Effects

The obtained results are presented in a structured format. For each organ system (blood, cardiovascular, gastrointestinal, kidney, liver, and lungs), we show a percentage indicating the probability (%) [15] of health effects due to 4-CMC, as summarized in Table 5.

Alongside these probabilities, the results highlighted specific atomic or functional groups in the structure (with toxicophores indicated in red). These groups were identified as contributing factors to the parameter values calculated for each organ system. This approach allows for a clearer understanding of the potential toxicological impact of the compound on different organs, based on its molecular structure. In particular, the gastrointestinal system and lungs showed the highest probabilities of adverse effects, of 90% and 80%, respectively. On the contrary, the cardiovascular system and blood showed moderate risks, with probabilities of 39% and 33%. The kidneys and liver were identified as the least affected, with probabilities of 15% and 17%.

#### 2.2.3. Genotoxicity

The Ames test is a widely used bacterial assay to detect mutagenicity, serving as an early indicator for potential carcinogenicity. A positive result in this test often suggests that a substance may have carcinogenic properties in living organisms [16]. This evaluation plays a crucial role in identifying and potentially excluding harmful substances during the early stages of research and development [17]. Results obtained for Ames test from various in silico tools are represented in Table 6.

The relatively low likelihood (45%) and a moderate reliable index (RI = 0.25) obtained with ACD/Labs Percepta do not suggest a potential risk of genotoxicity. Similarly, the VEGA application also concluded that the substance tested likely does not cause genotoxicity, although it did not provide an estimation of probability. Additionally, both OCHEM and ADMETlab categorized 4-CMC as not inducing genotoxic changes. OCHEM indicated an 80% probability of inactivity, while ADMETlab estimated an 88% chance of no genotoxicity. These findings are significant in toxicology, especially concerning the prolonged use of compounds like 4-CMC.

#### 2.2.4. Eye and Skin Irritation

Assessing the potential for eye and skin irritation is crucial to the safety of people who interact with substances made for industrial, pharmaceutical, or cosmetic purposes, as well as with NPSs. For six decades, Draize rabbit tests for eye and skin irritation have been used to predict how these products might irritate the human eyes and skin [18]. It should be noted that the models for the prediction of eye and skin irritation use data from more than 2000 compounds and provide comparisons with compounds with similar structures, enhancing the reliability of the predictions for compounds intended for topical application. The results of the eye and skin irritation potential of the examined compound are presented as probabilities (%) of severe or moderate irritation. The probability of causing eye irritation was 57%, suggesting a moderate probability of irritation. For skin irritation, the probability was higher, being 74%, indicating a substantial likelihood of the compound causing skin irritation. These results are significant to understand the irritative properties of 4-CMC and are critical for clinical and forensic purposes. The VEGA method provided opposite results, indicating a higher possibility of eye irritation than of skin irritation. However, those results seem to be less reliable, due to them being out of the applicability domain. Table 7 represents results obtained for eye and skin irritation from different softwares.

#### 2.2.5. Cardiotoxicity

Cardiotoxicity associated with the inhibition of the human ether-a-go-go (hERG) potassium channel is an increasingly common cause of attrition among drug candidates [19], including NPSs. The hERG channel plays a crucial role in normal cardiac depolarization, and its blockade can lead to the prolongation of the cardiac QT interval, thus increasing the risk of life-threatening arrhythmias [20]. The estimation of the half-maximal inhibitory concentration (IC50) is critical for assessing the potential cardiotoxic effects of drug-like compounds such as 4-CMC. Specifically, it was found that a concentration of 240.2 µM of 4-CMC is required to achieve a 50% inhibition of hERG channel activity. For hERG inhibition with a Ki value below 40 µM, ADMETlab assessed the likelihood to be no more than 40%, as shown in Table 8.

**Figure 2:**
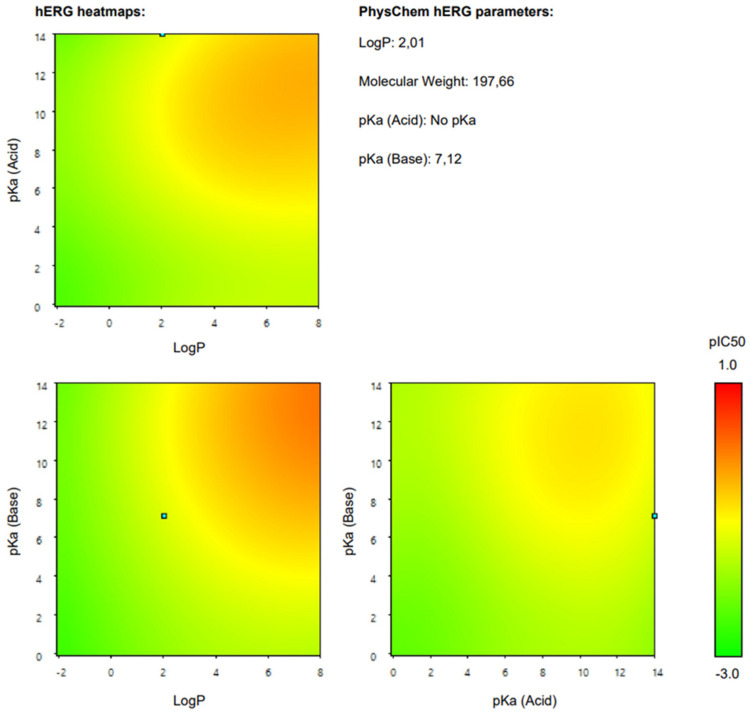
hERG heatmaps and physico–chemical hERG parameters for 4–chloromethcathinone (4–CMC).

Additionally, we present a heatmap of the obtained results illustrating the dependence of hERG inhibition potential on lipophilicity and ionization (Figure 2).

The heatmap specifically illustrates the partial dependency of the inhibition potential of hERG on the two pharmacokinetic parameters lipophilicity (logP) and ionization (pKa, base) in relation to 4-CMC. This analysis is crucial to understanding the cardiac safety profile of 4-CMC, given the importance of hERG channels in cardiac function. For 4-CMC, the most significant observation from the heatmap is the peak hERG inhibition potential at a logP value of 8 when considering the pKa (base).

On the other hand, 4-CMC as a potential hERG inhibitor is characterized by a Ki value less than 10 µM when tested using the patch clamp method. Based on the results obtained (Table 8), the probability of 4-CMC inhibiting the hERG channel at clinically relevant concentrations was estimated to be 3%, with moderate reliability (RI = 0.55).

#### 2.2.6. Endocrine System Disruption

This evaluation focused on estimating the interaction of the compound with the estrogen receptor, specifically, estrogen receptor alpha (ER-α), which is a key component of the endocrine system and plays a crucial role in hormonal regulation. The results of the evaluation of the disruption of the endocrine system by 4-CMC are presented in Table 9.

The probability of 4-CMC showing LogRBA values greater than −3, which would indicate some level of affinity for estrogen receptor alpha (ER-α), was calculated to be 0.03. The reliability of this prediction was considered moderate (RI = 0.62). On the other hand, the probability that 4-CMC exhibits LogRBA values greater than 0, which would indicate a stronger binding affinity for ER-α and potential estrogenic activity, was calculated to be 0.00; the reliability of this prediction was rated as high (RI = 0.79).

## 3. Discussion

The acute toxicity profile of 4-CMC revealed in this study provides critical information on its potential risks and biological interactions. The data indicate species-specific variations in toxicity. The relatively low LD_50_ values for the intravenous and intraperitoneal routes in this study are consistent with the high toxicity potential of synthetic cathinones when introduced directly into the bloodstream. The high toxicity observed with certain administration routes, particularly the intravenous one, is alarming, given the abuse patterns associated with NPSs. The reliability index (RI) values indicate confidence in the LD_50_ values, with higher values suggesting greater reliability. Most values fell in the moderate toxicity range, which requires cautious interpretation. Based on the results obtained, the most reliable value was estimated for mice following intraperitoneal administration (LD_50_ = 260 mg/kg; RI = 0.83, high reliability), suggesting that these data are the most useful. The other values obtained cannot be considered reliable (moderate reliability) because they were characterized by low RI values (in the range of 0.58–0.71). The TEST software employs multiple methods for predicting toxicity, leading to varying results. These methods include hierarchical clustering, Ward’s method, the nearest-neighbor method, and a consensus model utilizing all QSAR methods. Hierarchical clustering estimates toxicity by averaging predictions from different models, while Ward’s method divides the training set into similar structural clusters [21]. The nearest-neighbor method predicts toxicity by averaging those of similar compounds in the training set. The consensus model, considered the most reliable, incorporates all QSAR methods for toxicity assessment [14]. Utilizing TEST for quantifying acute toxicity provided values of LD_50_ ranging from 169.67 mg/kg bw (hierarchical clustering for oral administration in rats) to 345.38 mg/kg bw (nearest-neighbor method for oral administration in rats), whereas the consensus method established an LD_50_ of 242.07 mg/kg bw. The LD_50_ value following oral administration in rats was assessed to be 340 mg/kg bw by ProTox 3.0, with a probability of 70.97%. On the other hand, VEGA provided, for this same species and administration route, an LD_50_ value of 201.85 mg/kg bw. Considering qualitative methods, acute toxicity was assessed with STopTox, obtaining a high probability of toxicity (82%) following oral administration, a moderate probability (67%) following dermal administration, and a moderate probability of lack of toxicity (50%) following inhalation. The admetSAR results confirmed toxicity when 4-CMC is administered orally, with a probability of 85.47%, while ADMETLab yielded a probability of 65.1%. In the literature, there are no experimental research results on the acute toxicity of 4-CMC and its analogues. The only reports available are related to incidents involving this substance, though often, it is not the only substance implicated [4]. The highest reported concentration of 4-CMC in the blood and vitreous humor were, respectively, 8542 and 9874 ng/mL [22].

Regarding the results of the report from the health effects module, it is notable that the highest probabilities of adverse effects were observed for the gastrointestinal system and the lungs, corresponding to 90% and 80%, respectively. This suggests that these organs are significantly more susceptible to the toxicity of 4-CMC. In both cases, the toxicophore was identified as the ‘CH3–NH–’ fragment, which does not seem specific or particularly characteristic. In contrast, the cardiovascular system and the blood appeared to be at moderate risk of toxicity, with probabilities of 39% and 33%. The kidneys and liver were identified as the least affected, with probabilities of 15% and 17%, indicating a lower likelihood of adverse effects in these organs. It should be underlined that the lower probabilities for the kidneys and liver, while comparatively reassuring, still warrant attention due to the essential functions these organs perform. This kind of risk prediction highlights the importance of a comprehensive assessment in NPS evaluations. In addition, this susceptibility gradient observed for different organ systems is crucial for risk assessment and management for forensic toxicology purposes.

In the context of forensic and clinical toxicology, the Ames test is a popular and essential assay to evaluate the mutagenic potential of compounds such as 4-CMC, which are often used chronically for many years. The results obtained from Percepta, indicating a probability of 45% of a positive Ames test, with a moderate reliability index (RI = 0.51), suggest a notable but not definitive risk of genotoxicity for the compound tested. This result, although not conclusive, indicates a potential for DNA damage and carcinogenicity, especially relevant for substances of long-term use such as 4-CMC. On the other hand, the results obtained from OCHEM, ADMETlab, and VEGA indicated a high probability of 4-CMC not being genotoxic. Overall, considering the long-term usage of 4-CMC, these findings underscore the importance of assessing the mutagenic risk of this compound, as prolonged exposure to it could increase its potential for DNA damage and carcinogenic effects.

The results of eye and skin irritation potential provided the probabilities for 4-CMC to cause irritation in the standard rabbit Draize test [18]. The probability of eye irritation was 57%, indicating a moderate risk, while the probability of skin irritation was 74%, suggesting a higher probability of causing skin irritation during cosmetic use or any exposure to human skin and eyes. In contrast, the VEGA method presented opposite findings, suggesting a greater likelihood of eye irritation compared to skin irritation. However, these findings appear less dependable, as they fall outside the applicable domain.

Cardiotoxicity associated with the inhibition of the human ether-a-go-go (hERG) potassium channel is an increasingly common cause of attrition among drug candidates, including novel psychoactive substances like 4-CMC. The hERG channel plays a crucial role in normal cardiac depolarization, and its blockade can lead to the prolongation of the cardiac QT interval, thus increasing the risk of life-threatening arrhythmias. The determination of the half-maximal inhibitory concentration (IC50) is critical for evaluating the potential cardiotoxic effects of druglike compounds such as 4-CMC. In the context of the inhibition of the hERG channel, the IC50 value of 240.2 µM for 4-CMC signifies that this NPS exhibits moderate inhibitory potency. It is essential to note that inhibition of the hERG channel is a significant concern in the context of new psychoactive substances such as 4-CMC, as it can lead to the prolongation of the QT interval on electrocardiogram (ECG) and to potentially hazardous cardiac arrhythmias. Compounds that show significant inhibition of the hERG channel, including NPSs, are typically considered potentially dangerous. Therefore, further investigations and analyses are imperative to assess the cardiotoxicity risk associated with this IC50 result and to make informed decisions in the development and regulation of this NPS.

The results obtained from the predicted heat map (Figure 2) suggest that 4-CMC exhibits its highest potential for hERG inhibition under conditions of high lipophilicity. A logP of 8 is exceptionally high, indicating that 4-CMC, with such lipophilicity, can easily integrate into lipid-rich environments such as cell membranes, which is a crucial factor for the interaction of the hERG channel. The pKa (base) parameter reflects the ionization potential of 4-CMC in its basic form. The heat map indicates that at a certain level of pKa (base), in conjunction with a high logP, there is a significant increase in the potential for inhibition of the hERG channel by 4-CMC. The exact value of pKa at which this occurs is not specified, but its synergistic effect with high logP is evident. This finding is particularly relevant for evaluating the cardiac safety of 4-CMC. Inhibition of the hERG channel can lead to cardiac arrhythmias, making it a key concern in the clinical toxicology of NPS such as 4-CMC. The interaction between high lipophilicity (logP) and a specific range of pKa (base) in maximizing hERG inhibition suggests that modifying these properties in 4-CMC could be a strategy to mitigate cardiac safety risks.

On the other hand, Percepta’s calculated probability that 4-CMC inhibits the hERG channel at clinically relevant concentrations is relatively low (3%). This percentage represents the probability that 4-CMC may have a detrimental effect on the hERG channel. However, it is crucial to remember that even a low probability of inhibition of hERG can pose a significant risk, given the potential severity of cardiac arrhythmias. On the other hand, results from ADMETlab suggests that there is higher chance (40%) of 4-CMC inhibiting hERG channel.

The probability of 4-CMC exhibiting LogRBA values greater than −3, which would indicate some level of affinity for estrogen receptor alpha (ER-α), was calculated to be 0.03. This value suggests that there is a relatively low likelihood that 4-CMC will have a strong binding affinity for ER-α, as LogRBA values below −3 are typically indicative of non-binding compounds. The moderate RI indicated a reasonable degree of confidence in this estimation. Furthermore, the probability of 4-CMC exhibiting LogRBA values greater than 0, which would indicate a stronger binding affinity for ER-α and potential estrogenic activity, was calculated to be 0.00. This result suggests that there is essentially no likelihood that 4-CMC will exhibit strong binding to the estrogen receptor. The high RI indicated a high level of confidence in this estimate, reinforcing the conclusion that 4-CMC is not likely to act as a strong estrogen receptor binder. These results indicate that 4-CMC is not expected to strongly bind to the estrogen receptor alpha (ER-α) and exhibit estrogenic activity. The low probability of LogRBA values exceeding 0, coupled with the high reliability of this prediction, supports the conclusion that 4-CMC is unlikely to exert significant endocrine-disrupting effects through strong estrogen receptor binding. It is important to consider these findings in the context of the potential impact of the compound on the endocrine system, particularly with respect to reproductive and hormonal regulation. Further studies and assessments may be necessary to fully understand the extent of 4-CMC endocrine-disrupting properties and its implications for human health.

In conclusion, the in silico prediction of toxicity for 4-CMC represents a transformative approach in both regulatory and medical fields. In the regulatory domain, in silico toxicity prediction provides authorities with a powerful tool for risk assessment and management. This facilitates the timely identification and restriction of hazardous substances before they proliferate in the market, thereby protecting public health and safety. Regulatory bodies can thus act more swiftly and efficiently, relying on robust computational data to guide policy and enforcement decisions. In the medical field, in silico models assist in understanding the toxicological profiles of NPSs, which is essential for developing therapeutic strategies and managing cases of intoxication. Physicians and toxicologists can utilize these predictions to better anticipate adverse effects, leading to improved patient outcomes through more informed treatment protocols.

While not a complete substitute for experimental testing, these computational models offer rapid, cost-effective, and ethical preliminary evaluations. Their growing accuracy and predictive power make them indispensable for early risk assessment, aiding regulatory bodies in making informed decisions and supporting medical professionals in managing NPS-related health issues. However, continued refinement and validation against empirical data are essential to fully establish their reliability and comprehensiveness in toxicity prediction.

## 4. Materials and Methods

### 4.1. Study Design

**Figure 3:**
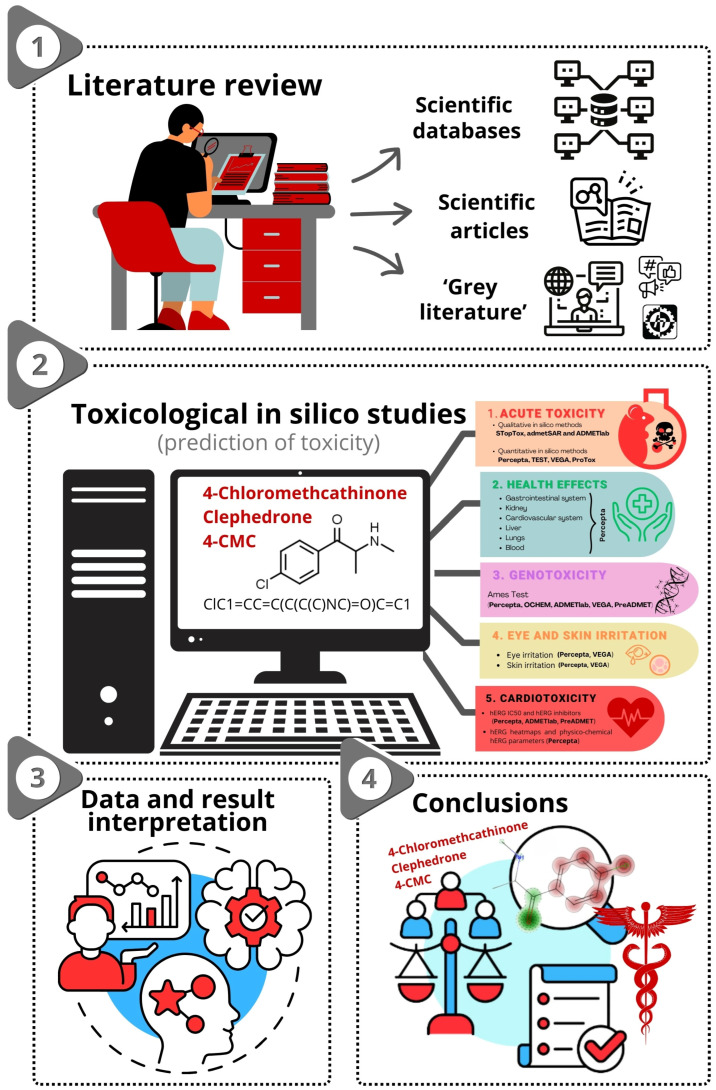
Study design for prediction of toxicity of 4-CMC.

The objective of this study was to analyze the toxicity of 4-CMC using various in silico simulation software applications. The study was designed to ensure consistency in input data and conditions across all simulations, providing a comprehensive evaluation of the toxicity profile of 4-CMC. Therefore to ensure consistency, for every utilized method, we decided to input 4-CMC SMILES as follows: ClC1=CC=C(C(C(C)NC)=O)C=C1. A conceptual overview of this study is represented in Figure 3.

### 4.2. Toxicity Endpoints

Considering that 4-CMC is an NPS, its use and exposure duration can vary widely. Due to the lack of any toxicological data on this substance, the focus of this study was on determining six key toxicity parameters: (1) acute toxicity; (2) health effects on various organs and systems (blood, cardiovascular system, gastrointestinal system, kidney, liver, lungs); (3) genotoxicity; (4) eye and skin irritation; (5) cardiotoxicity; (6) endocrine system disruption. These parameters were selected to provide a comprehensive overview of the toxicological profile of 4-chloromethcathinone, providing information on its safety and the risks associated with its use. The estimation of these key toxicity parameters is of significant importance in the fields of forensic and clinical toxicology, particularly given the absence of previous research data on this NPS. The rationale for selecting these specific parameters is rooted in their crucial relevance to understanding both the toxic profile of the substance and its applicability in forensic and clinical toxicology settings.

- Acute toxicity and health effects: the prediction of acute toxicity and health effects is vital in assessing the immediate and long-term risks associated with exposure to 4-CMC; in forensic toxicology, understanding acute toxicity helps determine the cause of acute poisoning incidents, while in clinical toxicology, it guides treatment strategies for overdose cases;
- Genotoxicity: assessing the genotoxic potential of 4-CMC is critical, as genetic mutations or DNA damage can have profound implications for human health; this is particularly relevant in forensic toxicology, to evaluate long-term exposure risks and potential carcinogenicity;
- Eye and skin irritation: the prediction of eye and skin irritation is crucial for understanding the risks of accidental exposure; this information is essential in both forensic and clinical toxicology, to assess cases of dermal or ocular exposure and to provide appropriate medical interventions;
- Cardiotoxicity: given the increasing concern about the cardiovascular effects of NPSs, evaluating the cardiotoxic potential of 4-CMC is essential; this parameter assists in assessing the risk of heart-related complications, which is significant both for emergency medical responses and for forensic investigations;
- Endocrine system disruption: investigating the potential of 4-CMC to disrupt the endocrine system is important due to the critical role that hormones play in bodily functions; endocrine disruption can lead to a range of health issues, making this parameter highly relevant in clinical assessments and forensic analysis.

### 4.3. Utilized In Silico Software

To ensure proper readability, in Table 10, we report the utilized software providing with a short description and indicating which toxicity endpoints were estimated using each software.

## Figures and Tables

**Table 1:** Physicochemical characterization of 4-chloromethcathinone (4-CMC, clephedrone), gathered from different databases [6,7].

Name(s)	4-Chloromethcathinone, Clephedrone
IUPAC name	1-(4-chlorophenyl)-2-(methylamino)-1-propanone
Acronym	4-CMC
Molecular formula	C10H12ClNO
Structural formula	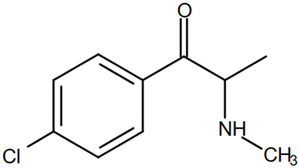
CAS	1225843-86-6
SMILES	ClC1=CC=C(C(C(C)NC)=O)C=C1
Molecular weight, g/mol	197.66
Density, g/mL	1.1
Melting point, °C	198

**Table 2:** The classification of 4-chloromethcathinone (4-CMC, clephedrone) from a forensic toxicology perspective in various regions, according to the regulation of therapeutic goods available from government websites.

Country/Region	Status
Germany	List I controlled drug
Sweden	Suggested as illegal narcotic (June 2015)
China	Controlled substance (as of October 2015)
Virginia, USA	Schedule 1 substance
USA	Schedule II (December 2019)
Poland	Listed among new psychoactive substances (since August 2018)

**Table 3:** Qualitative in silico prediction of toxicity and eye/skin irritation for 4-CMC using STopTox, admetSAR, and ADMETlab.

Type of Acute Toxicity	STopTox (https://stoptox.mml.unc.edu/, accessed on 30 April 2023)				AdmetSAR 3.0		ADMETlab 2.0
	Prediction	Confidence (%)	Applicability Domain	Predicted Toxicophore(s) *	Classification	Probability (%)	Probability of Being Toxic
Oral acute toxicity	Toxic (+)	82.0	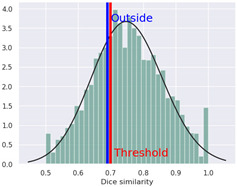	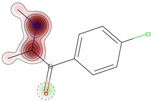	III	85.47	0.651
Dermal acute toxicity	Toxic (+)	67.0	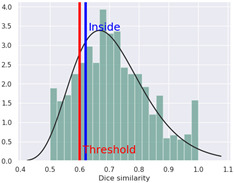	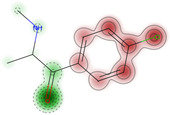	NA	NA	NA
Inhalation acute toxicity	Non-Toxic (−)	50.0	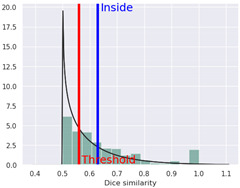	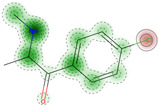	NA	NA	NA
Eye irritation	Negative (−)	62.0	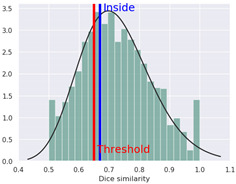	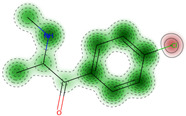	−	58.38	NA
Skin irritation	Negative (−)	80.0	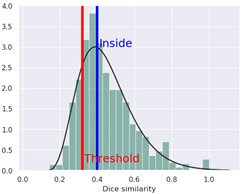	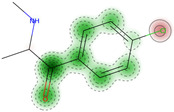	+	53.28	NA

* Structural fragments that increase toxicity are highlighted in red, whereas those that lower toxicity are highlighted in green. NA—not applicable.

**Table 4:** Results of the prediction of acute toxicity for 4-chloromethcathinone (4-CMC, clephedrone).

Software	Species	Administration Route	LD50, mg/kg bw.	Reliability/Similarity Coefficient
Percepta 2023.1.2	Rat	Oral	470.00	Moderate, RI = 0.65
		Intraperitoneal	330.00	Moderate, RI = 0.58
	Mouse	Oral	620.00	Moderate, RI = 0.61
		Intraperitoneal	260.00	High, RI = 0.83
		Intravenous	120.00	Moderate, RI = 0.70
		Subcutaneous	310.00	Moderate, RI = 0.71
TEST 5.1.2. (Consensus)	Rat	Oral	242.07	SC ≥ 0.9
TEST 5.1.2. (Hierarchical clustering)	Rat	Oral	169.67	SC ≥ 0.9
TEST 5.1.2. (Nearest neighbor)	Rat	Oral	345.38	SC ≥ 0.9
VEGA QSAR 1.2.3	Rat	Oral	201.85	SC ≥ 0.9
ProTox 3.0	Rat	Oral	340.00	SC = 70.97%

RI—reliability index; SC—similarity coefficient.

**Table 5:** Results of the health effect prediction for 4-chloromethcathinone (4-CMC, clephedrone).

Health Effect	Probability of Health Effects, %	Atomic/Functional Group Contributions to the Calculated Parameter Values	Details
Gastrointestinal system	90	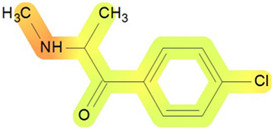	Toxic effects: nausea or vomiting Changes in structure or function of salivary glands Route: oral Species: human, female; rat; dog Studies: acute; chronic
Lungs	80	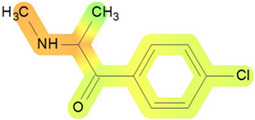	Toxic effects: dyspnea Route: oral Species: rat; mouse Studies: acute
Cardiovascular system	39	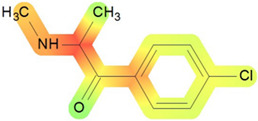	Toxic effects: pulse rate increase without fall in blood pressure and other changes Route: oral Species: human, male and female Studies: acute
Blood	33	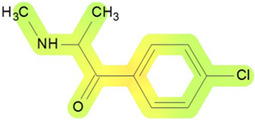	No specific values given
Liver	17	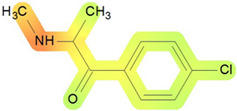	Toxic effects: changes in liver weight Route: oral Species: rat Studies: chronic
Kidney	15	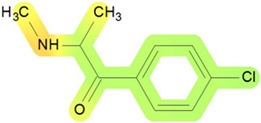	Toxic effects: changes in bladder weight and other changes Route: oral Species: rat Studies: chronic

**Table 6:** Results of genotoxicity. Ames test effect prediction for 4-chloromethcathinone (4-CMC, clephedrone).

Predicted Ames Test Results	Probability (%)	Structure with Toxicophores	Software
Mutagen	45 (Moderate reliability, RI = 0.51)	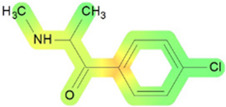	Percepta 2023.1.2
Inactive	80	N/A	OCHEM (https://ochem.eu/predictor/show.do, accessed on 30 April 2023)
Nontoxic	88		ADMETlab 3.0
Non-mutagen	N/A		VEGA QSAR 1.2.3

RI—reliability index; AD—applicability domain; N/A—not applicable.

**Table 7:** Results of eye and skin irritation prediction for 4-chloromethcathinone (4-CMC).

Eye Irritation	Skin Irritation	Software
Probability: 57%	Probability: 74%	Percepta 2023.1.2
Irritant (out of AD)	Not irritant (possibly out of AD)	VEGA QSAR 1.2.3

AD—applicability domain.

**Table 8:** Results of cardiotoxicity prediction, hERG IC50 and hERG inhibition, for 4-chloromethcathinone (4-CMC).

Cardiotoxicity Predictions	Probability	Reliability	Software
hERG half-maximal inhibitory concentration	IC50 = 240.2 µM	N/A	Percepta 2023.1.2
hERG inhibition (Ki < 10 µM, patch clamp)	3%	Moderate (RI = 0.55)	Percepta 2023.1.2
hERG inhibition (Ki < 40 µM, patch clamp)	40%	N/A	ADMETlab 3.0

RI—reliability index; N/A—not applicable.

**Table 9:** Results of the prediction of endocrine disruption by 4-chloromethcathinone (4-CMC).

Probability of Estrogen Receptor Binding			
LogRBA > −3	Reliability	LogRBA > 0	Reliability
0.03	Moderate (RI = 0.62)	0.00	High (RI = 0.79)

RI—reliability index.

**Table 10:** Utilized in silico software.

Software	Description	Toxicity Endpoints
StopTox (https://stoptox.mml.unc.edu/, accessed on 30 April 2023)	STopTox is a sophisticated software designed to predict human acute toxicity tests known as the ‘6-pack’ [10]. These tests cover various aspects such as oral, dermal, and inhalation toxicity, as well as irritation and sensitization [23]. The predictions are based on quantitative structure–activity relationship (QSAR) models validated using animal experimental data. The machine learning algorithm, random forest, along with MACCS descriptors, is employed for prediction. It offers a swift screening method for assessing chemical toxicity, identifying molecular components that increase or decrease toxicity, and providing fragment contribution predictions.	Qualitative: acute toxicity, skin and eye irritation.
AdmetSar 3.0	AdmetSAR has emerged as a comprehensive software solution for predicting the ADMET (absorption, distribution, metabolism, excretion, and toxicity) properties of chemicals [24,25]. Its latest version, admetSAR 3.0, integrates over 40 predictive models using in silico filtering techniques to evaluate new chemical properties related to ADMET [26,27]. The model uses a dataset of 10,207 molecules with LD_50_ values determined in rat [28].	Qualitative: oral toxicity, skin and eye irritation
ACD/Labs Percepta 2023.1.2	The ACD/Labs Percepta platform is commercially available scientific software for predicting different toxicological properties using computational methods. It provides insight into how chemical structures correlate with a wide array of ADME, toxicological, and physicochemical characteristics. The platform includes a structure optimization module with comprehensive ADMET filters and takes advantage of various data inputs to generate predictions. The reliability and accuracy of Percepta models are continually evaluated and refined based on new scientific data. Research was carried out in Percepta version 2023.1.2 with a license purchased by the Institute of Medical Expertises in Łódź.	Quantitative: acute toxicity (LD_50_), health effects (blood, cardiovascular system, gastrointestinal system, kidneys, liver, lungs), genotoxicity (mutagenicity as Ames test), eye and skin irritation, cardiotoxicity (hERG inhibition), endocrine system disruption.
ProTox 3.0	ProTox 3.0, updated scientific software released in March 2024, utilizes diverse machine learning models and databases to predict toxicological properties [29,30]. Mainly applied in drug development and chemical safety evaluation, ProTox 3.0 provides valuable insights into the potential toxic effects of novel compounds. Predictions include similarity- and fragment-based approaches, generating alerts for potential toxicity targets, with results provided with confidence scores, a comprehensive toxicity radar chart, and details on analogous known toxic compounds.	Quantitative: acute toxicity
ADMETlab 2.0	ADMETlab 2.0 is an expansive scientific platform dedicated to predicting the ADMET properties of compounds [31,32]. Designed for user-friendliness and efficiency, ADMETlab 2.0 facilitates batch computation and features an intuitive interface. The prediction models utilize graph-based neural networks with an attention mechanism, producing precise and nuanced predictions across various ADMET-related endpoints.	Qualitative: acute toxicityQuantitative: genotoxicity (mutagenicity by Ames test), cardiotoxicity (hERG inhibition)
OCHEM (https://ochem.eu/predictor/show.do, accessed on 30 April 2023)	The online chemical modeling environment (OCHEM) is a web-based platform strategically crafted to simplify and automate traditional processes in quantitative structure–activity relationship (QSAR) modeling [33,34]. This database is intricately linked with the modeling framework, which assists users through all necessary steps in developing a predictive model: data retrieval, calculation and selection of various molecular descriptors, application of machine learning methods, validation, model analysis, and assessment of the applicability domain [35,36].	Quantitative: genotoxicity (mutagenicity as Ames Test)
TEST 5.1.2	The toxicity estimation software tool (TEST) was created as an open-source application by the U.S. EPA [37,38,39]. The software utilizes various molecular descriptors sourced from the EPA ECOTOX databases. TEST employs three QSAR approaches: the hierarchical method groups compounds based on structural similarities [21], the nearest-neighbor method averages values from structurally similar chemicals [40], and the consensus method combines predictions from all preceding methods [41].	Quantitative: acute toxicity
VEGA QSAR 1.2.3	VEGA QSAR, a collaborative effort from EU projects, integrates QSAR models and rule-based expert systems to predict various human toxicity endpoints [42]. The platform includes an applicability domain index for reliability assessment and conducts automatic checks to identify potential issues that could compromise prediction accuracy or reliability. These checks include verifying similarity between the target compound and those in the dataset, assessing concordance between experimental values and predictions, evaluating precision, and more. VEGA’s comprehensive approach ensures robust and reliable toxicity predictions, aiding in risk assessment.	Quantitative: acute toxicity, genotoxicity (mutagenicity by Ames test), eye and skin irritation.

## Data Availability

The data, analytic methods, and study materials that support the findings of this study are available from Kamil Jurowski (toksykologia@ur.edu.pl) on reasonable request.

## Abbreviations

4-CMC	4-Chloromethcathinone
ADME	Absorption, distribution, metabolism, and excretion
hERG	Human ether-a-go-go-related gene
IC_50_	Half-maximal inhibitory concentration
LD_50_	Median lethal dose rate 50
LogP	Logarithm of the partition coefficient
LogRBA	Logarithm of relative binding affinity
NPSs	New psychoactive substances
pKa	Acid dissociation constant
RI	Reliability index
QSAR	Quantitative structure–activity relationships
